# Poloxamers Have Vaccine-Adjuvant Properties by Increasing Dissemination of Particulate Antigen at Distant Lymph Nodes

**DOI:** 10.3390/molecules28124778

**Published:** 2023-06-15

**Authors:** Myriam Lamrayah, Capucine Phelip, Renaud Rovera, Céline Coiffier, Nora Lazhar, Francesca Bartolomei, Evelyne Colomb, Bernard Verrier, Claire Monge, Sophie Richard

**Affiliations:** 1 Laboratory of Tissue Biology and Therapeutic Engineering, Institut de Biologie et Chimie des Protéines, UMR 5305, CNRS/Claude Bernard University Lyon 1, 7 Passage du Vercors, CEDEX 07, 69367 Lyon, France; myriam.lamrayah@epfl.ch (M.L.); cp@hephaistos-pharma.com (C.P.); renaud.rovera@ibcp.fr (R.R.); celine.coiffier@ibcp.fr (C.C.); nora.lazhar01@gmail.com (N.L.); bartolomei.franc@gmail.com (F.B.); evelyne.colomb@ibcp.fr (E.C.); bernard.verrier@ibcp.fr (B.V.); sophie.richard@ibcp.fr (S.R.); 2 Laboratory of Virology and Genetics, School of Life Sciences, Ecole Polytechnique Fédérale de Lausanne (EPFL), 1015 Lausanne, Switzerland

**Keywords:** poloxamer, vaccine, adjuvant, subcutaneous, lymph node, nanoparticles

## Abstract

Vaccine technology is still facing challenges regarding some infectious diseases, which can be addressed by innovative drug delivery systems. In particular, nanoparticle-based vaccines combined with new types of adjuvants are actively explored as a platform for improving the efficacy and durability of immune protection. Here, biodegradable nanoparticles carrying an antigenic model of HIV were formulated with two combinations of poloxamers, 188/407, presenting or not presenting gelling properties, respectively. The study aimed to determine the influence of poloxamers (as a thermosensitive hydrogel or a liquid solution) on the adaptive immune response in mice. The results showed that poloxamer-based formulations were physically stable and did not induce any toxicity using a mouse dendritic cell line. Then, whole-body biodistribution studies using a fluorescent formulation highlighted that the presence of poloxamers influenced positively the dissemination profile by dragging nanoparticles through the lymphatic system until the draining and distant lymph nodes. The strong induction of specific IgG and germinal centers in distant lymph nodes in presence of poloxamers suggested that such adjuvants are promising components in vaccine development.

## 1. Introduction

Vaccination can be defined as a means of preparing the body’s immune system for the rapid elimination of infectious agents or their toxic products. It is one of the most effective medical interventions, and it has been estimated—according to the Center for Disease Control and Prevention—to have prevented more than 50 million deaths worldwide between the years 2021 and 2030 [1]. The eradication of smallpox in 1978 [2] and the most up-to-date successful emergence of mRNA vaccines, significantly blunting the impact of the COVID-19 pandemic, are proof of their importance [3]. However, vaccine technology still faces ongoing challenges in terms of efficacy, practicality, and safety [4]. Several infectious diseases cannot yet be avoided by protective vaccination (HIV, HCV), and most of the existing vaccines require multiple injections of complex antigens (Ag) or must be stored under restrictive conditions, some examples of substantial limitations of use. Another major hurdle is eliciting a sufficiently potent immune response while meeting the safety standards necessary for prophylactic vaccines designed for administration to healthy (often infant) populations or immunocompromised persons (cancerous or elderly), as exemplified by the different difficulties encountered with influenza vaccines [5].

Most licensed human vaccines rely on antibody (Ab)-mediated responses for protection, but recent studies have highlighted the primary role of CD4+ T cells and germinal centers (GCs) in lymph nodes (LN) for the elicitation of such Abs, placing strong importance on the crosstalk between B cells, follicular dendritic cells (FDCs), and T follicular cells (Tfh) as well as the presence of a “native Ag” [6,7]. Indeed, soluble Ag can lose native structure due to lability or proteolysis over time (leading to exposure of irrelevant/distracting epitopes), whereas a sustained release will ensure the availability of an intact immunogen, favoring immune responses against native forms/epitopes of the protein [8]. One of the most recent approaches used to enhance the immunogenicity of subunit vaccines is Ag formulation with biomaterial-based particulate carriers [9]. Thus, we hypothesize that formulating vaccine Ag onto the surface of a nanoparticle (NP) will stabilize its native form and prevent its degradation by proteases [10,11]. Various studies have demonstrated that Ag loading into synthetic particles enhances uptake by presenting cells, such as dendritic cells (DCs), as well as by increasing Ag bioavailability by promoting retention in secondary lymphoid organs [12,13,14]. Furthermore, these particulate carriers are able to co-deliver adjuvants, mimicking microbial danger signaling [15]. Our group has already shown that co-delivery of vaccine Ag loaded onto 200–300 nm polylactic acid (PLA) NPs in the presence of different pattern recognition receptors (PRR) ligands or chimeric toll-like receptors (TLR)/nucleotide oligomerization domain (NOD) ligands could amplify immune responses after subcutaneous administration [11,16].

NP should be larger than 20 nm to be transported through lymphatic vessels instead of travelling through the blood stream. The lymphatic draining of NP larger than 100 nm can take several days [17]. To improve the delivery of our nanoparticulate vaccine candidate, we took advantage of U.S.-Food-and-Drug-Administration-approved poloxamer gelling agents, used historically in pharmaceutical science as excipients for their different mechanical properties (mucoadhesion, lubrication, thermoreversibility) [18,19] and more recently in nanotherapeutic engineering [20]. Indeed, they provide increased solubility and stability to particulate formulations, permitting complex administrations of molecules or vaccines [21,22,23]. Poloxamers are a class of water-soluble nonionic triblock co-polymers formed by polar (polyoxyethylene) and nonpolar (polyoxypropylene) blocks. This coexistence of hydrophilic and hydrophobic monomers into blocks confers amphiphilic properties and allows the formation of ordered structures in solution, such as micelles. Through the embedding of NPs, diffusion and cellular uptake are thus improved [24,25]. Moreover, it is evidenced that the targeting the tumor-draining LN for the transportation of an immunotherapeutic adjuvant was achieved by the use of poloxamer P407 (Pluronic F127) [26] and that it could be used for lymphography [17].

During an infection process, the organism is in constant contact with the pathogen, allowing Ag-presenting cells to integrate all request signals for initiating a strong immune response [27]. This contact window is largely reduced when subunit vaccines are used, and it is likely that some immune actors could have been bypassed or simply absent from the injection site, leading to a suboptimal immune response [28]. Even if the use of adjuvants could increase their immunogenicity, most subunit vaccines require repeated injections (prime–boost strategy) to reach an efficient level of protection. It seems reasonable to assume that a sustained immune response could be also dependent on continuous stimulation by the vaccine Ag [29]. Poloxamer-based thermosensitive hydrogels have already proven to be efficient in controlling the delivery kinetics of anti-tumor blocking antibodies [30].

To evaluate if the use of poloxamers in vaccine formulation could enhance the adaptive immune response, we produced a dual delivery system designed to provide both (i) protection of the Ag through the NP vectorization and (ii) facilitated transport to LN through the lymphatic system after subcutaneous administration [31]. We have evaluated safe-by-design, non-immunogenic, and biocompatible formulations made of poloxamers P188 and P407 associated with biodegradable NPs of PLA carrying HIV-1 p24 protein as an antigenic model. These poloxamer-based formulations containing NPs constituted a vaccine inoculum that was easy to inject. The NP biodistribution induced by a hydrogel-forming formulation was compared to a non-gelling poloxamer formulation. Our objectives were to investigate (i) the impact of poloxamer gelation on the Ag residence time at the injection site and (ii) track the LN targeting to evaluate the subsequent adjuvant properties of poloxamers.

## 2. Results

### 2.1. Poloxamer Formulations as Liquid or Thermosensitive Hydrogel

**Figure 1:**
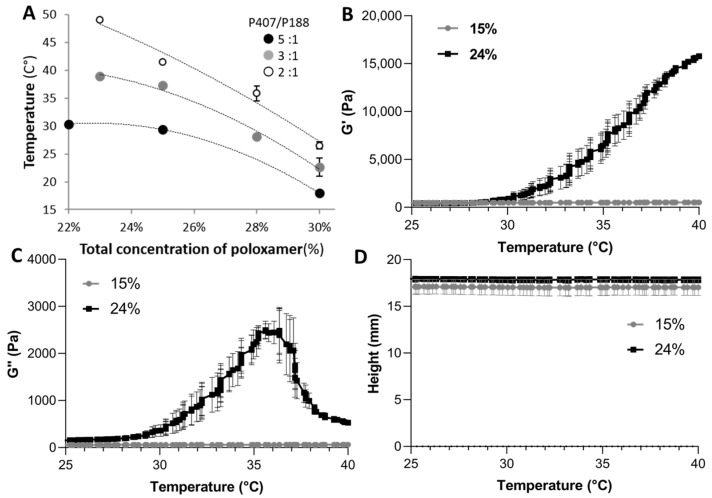
Rheological characterization of the poloxamer formulations as a function of the poloxamer concentration. (**A**) Sol–gel transition temperature (T_sol/gel_) at different P407 and P188 ratios and concentrations. Elastic (storage) modulus G′ (**B**) and viscous (or loss) modulus G″ (**C**). (**D**) Height of the poloxamer formulations during the temperature ramp. Error bars are represented as SD.

Rheological analysis was performed to evaluate the sol–gel transition temperature (T_sol/gel_) for different poloxamer formulations according to different P407:P188 ratios (5:1, 3:1, and 2:1, *w*/*w* ratio) and poloxamer concentrations (from 22 to 30% in *w*/*v* ratio). T_sol/gel_ was found in a range from 17 to 50 °C (Figure 1A). For efficient gelation at body temperature, an intermediate ratio of 4:1 and a concentration of 24% were selected, showing a gelation process starting around 30 °C in our experimental conditions, as emphasized by the increase in the storage (elastic) modulus G′ (Figure 1B).

**Figure 2:**
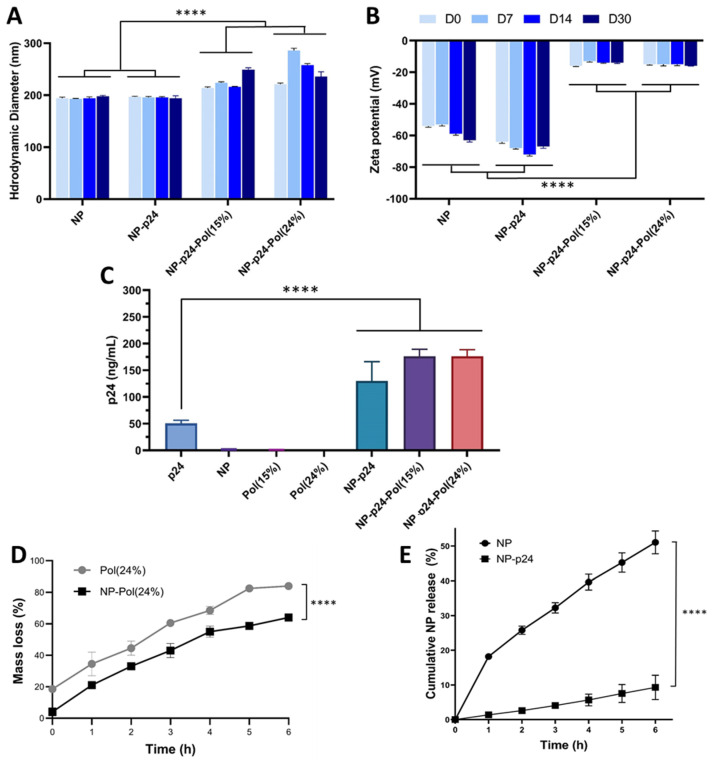
Characterization of NP formulation alone, with p24, and with poloxamers. In vitro stability of NP, NP-p24, NP-p24-Pol24%, and 15% formulations. The hydrodynamic diameter (nm) (**A**) and zeta potential (mV) (**B**) of the NP were measured via dynamic light scattering at day 0, 7, 14 and 30 after production. (**C**) Amount of p24 assayed (ng/mL) using sandwich ELISA. (**D**) In vitro stability of hydrogel (24% 4:1) with or without NP at 37 °C with PBS buffer (pH 7.4). (**E**) NP-p24 release from hydrogel (24% 4:1) over 6 h in presence and absence of p24 antigen. Data are presented as mean ± SD and were statistically analyzed using two-way ANOVA, followed by Tukey’s multiple comparison tests for (**A**–**C**) (**** *p* < 0.0001). Pol: poloxamer.

The T_sol/gel_ of the 24% thermosensitive hydrogel was determined to be 35–36 °C. This 24% poloxamer formulation was compared to a 15% poloxamer formulation with an identical P407:P188 ratio of 4:1, which has no gelation properties (Figure 1B). The determination of the loss (viscous) modulus G″ (Figure 2C) indicated that the 24% formulation has a transient shift in viscosity. As the tan δ (representing the measure of the viscoelastic balance of the behavior of the material) remained below 1 during all the acquisitions, we can assume that the 24% poloxamer formulation has predominantly gel-like elastic behavior [32]. The stable height of the formulations during the temperature ramp indicated the absence of physical contraction of the formulations (Figure 1D).

### 2.2. Incorporation of Nanoparticles Carrying a Protein Ag

NP suspensions were prepared via nanoprecipitation and characterized using dynamic light scattering (DLS) (Table 1).

The NPs alone had an average hydrodynamic diameter of 194 nm. After the addition of p24 to the surface of the NPs, the average hydrodynamic diameter reached 197 nm. The formulations with poloxamers induced significant increases in hydrodynamic diameter of up to 214 nm and 221 nm for 15% and 24%, respectively.

Concerning the zeta potential at D0, the NP alone had a negative charge of around −54 mV. After the adsorption of p24, the latter became more negatively charged, with a value of around −64 mV. The addition of the poloxamers (at 15% and 24%) induced a considerable increase in charges (around −16 and −14 mV respectively).

The addition of each element (p24 or poloxamers) induced an increase in the hydrodynamic diameter, while the poloxamers induced a significant increase in the surface charges of the formulations. These changes in physicochemical properties confirmed the efficiency of the p24 adsorption step and the addition of the poloxamer crown.

Characterization of NP alone, with NP-p24, and after the addition of poloxamers was carried out to measure the hydrodynamic diameter and the surface charge of NP over time (Figure 2A,B). This follow-up was performed over 30 days (D0, D7, D14, D30). The stability study highlighted no substantial variations of the hydrodynamic diameter or zeta potential over time regardless of the formulation.

The sandwich ELISA was used to determine the amount of p24 present in the samples (Figure 2C). The amount of p24 alone was determined to be around 50 ng/mL, while the amounts of p24 for the NP-p24, NP-p24-Pol(15%), and NP-p24-Pol(24%) conditions were significantly higher than for the p24 alone conditions (between 150 and 200 ng/mL). This was explained by the adsorption of multiple p24 proteins to each NP, determined to be around nine molecules of p24 per NP (estimation from NP concentration, p24 concentration and NP diameter).

The presence of p24 around NP decreased the amount of 24% hydrogel mass loss over time without modifying the general trend of degradation kinetics compared to the sample containing empty NP (Figure 2D). Moreover, the presence of a p24 corona around the NP significatively slowed down the p24 release from the 24% hydrogel (Figure 2E).

### 2.3. Toxicity and Activation of Immune Receptors

**Figure 3:**
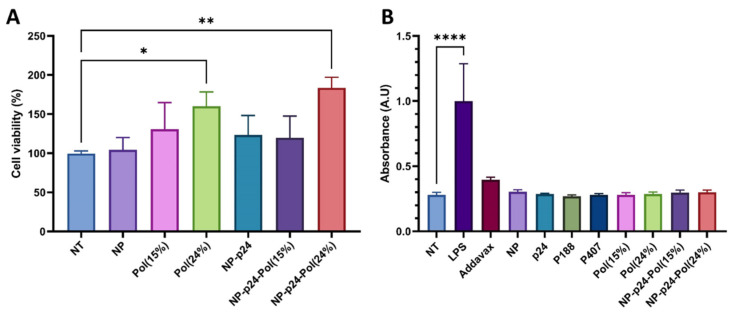
(**A**) Cell viability of DC2.4 cells following contact for 24 h with formulations (NP-p24, NP-p24-Pol(24%) and (15%)). (**B**) Quantification of NFκB pathway activation of RawBlue™ reporter cells after the addition of different formulations and their components. NT: not treated, NP: nanoparticles, Pol(X%): poloxamers at a concentration of X%, LPS: lipopolysaccharide. Data are presented as mean ± SD and were statistically analyzed using two-way ANOVA followed by Dunnett’s multiple comparison tests (* *p* < 0.1, ** *p* < 0.01, **** *p* < 0.0001).

Cell viability of DC2.4 cells, an immortalized murine dendritic cell line, was measured for the different formulations used (Figure 3A). NP alone and NP-p24 did not show a significant difference compared to the viability of untreated cells (NT). Similar results were obtained for the conditions with the 15% hydrogel (Pol(15%) and NP-p24-Pol(15%)). In contrast, cell viability measured in the presence of 24% hydrogel (Pol(24%) and NP-p24-Pol(24%)) was significantly higher (160% and 184%, respectively) than in the control conditions (NT), possibly explained by increased viscosity at 37 °C favoring cell adhesion and proliferation.

Activation of the NFκB pathway through activation of receptors of the innate immunity was studied in RawBlue™ murine macrophages for all formulations and their components (Figure 3B). Lipopolysaccharide (LPS), capable of binding to TLR4 (positive control), was able to activate the NFκB pathway. In contrast, none of the tested formulations activated innate receptors.

### 2.4. Whole-Body Biodistribution

**Figure 4:**
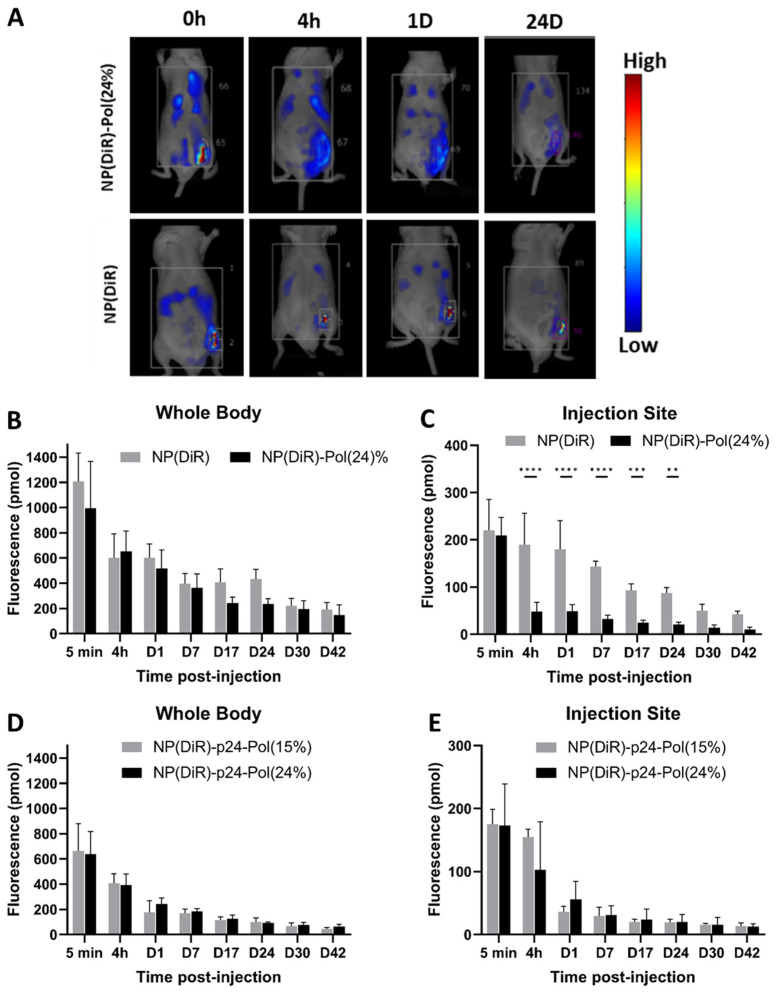
In vivo visualization of NP(DiR) in poloxamer formulations in the whole bodies and injection sites of mice. (**A**) Fluorescence imaging for in vivo tracking NP(DiR) with or without hydrogel (Pol(24%)). (**B**) Quantitative analysis of fluorescence intensity in the whole body. (**C**) Quantitative analysis of fluorescence intensity at the injection site. (**D**) Quantitative analysis of fluorescence intensity after NP-p24-Pol at 15% or 24% in the whole body (**E**) and at the injection site. Data are presented as mean ± SD and were statistically analyzed using two-way ANOVA (** *p* < 0.01, *** *p* < 0.001, **** *p* < 0.0001).

The kinetics of fluorescent NP biodistribution in the whole body of mice were investigated using fluorescence molecular tomography. After subcutaneous injection (above the left inguinal LN), we compared the quantity of fluorescence in the whole body and at the injection site for two formulations—NP(DiR) and NP(DiR)-Pol(24%) (Figure 4A)—over 42 days.

The DiR quantity was similar for each injection. The biodistribution study showed that in both NP groups, the fluorescence intensity within the whole body was similar up to 42 days after injection. We observed the same degradation profile of fluorescence in the whole body for both formulations, all along the experiment (Figure 4B). However, interestingly, we observed an important difference in fluorescence intensity at the injection site from 4 h after injection (Figure 4C). For NP (DiR)-Pol(24%), only 25% of the starting quantity remained at the injection site. This is contrary to the NP (DiR) group, which showed a progressive and slow profile of fainting from this site, with around 90% of the initial fluorescence detectable at the injection site during the first 24 h.

To understand the impact of gelation on the biodistribution of the formulations in the presence of the vaccine Ag, the NP (DiR)-p24 formulation was added to the poloxamer mixture at 24% (with a T_sol/gel_ around 35–36 °C) and 15% (without gelation). After subcutaneous injection of these formulations, the fluorescence intensity in the whole body and at the injection site was measured. No difference in biodistribution was observed (Figure 4D,E), highlighting that concentrations of poloxamer of 15% or 24%, and thus subsequent gelation, did not modify the biodistribution of NPs. We hypothesized that the presence of poloxamers themselves, and not the gelation process, influenced the dissemination of the NP.

### 2.5. Distribution of NPs in the Draining and Distant Lymph Nodes

**Figure 5:**
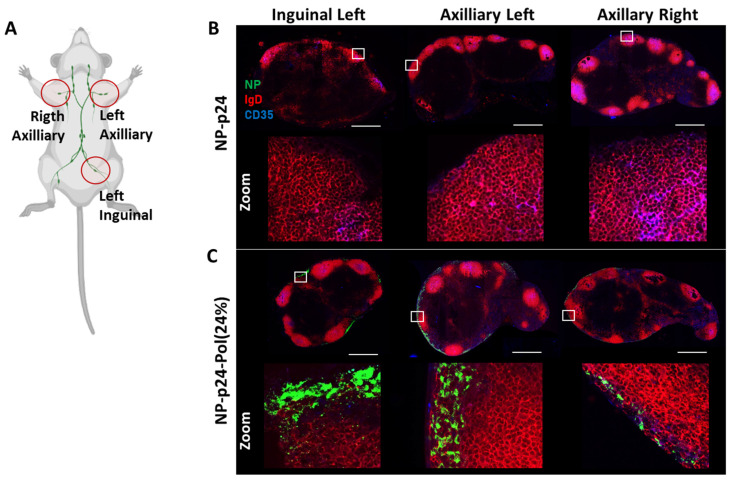
Distribution of NPs (BodipyFL)-p24 within the draining and distant LNs according to the absence or presence of poloxamers. (**A**) Schematic representation of the location of inguinal and axillary LNs in the mouse. Created with BioRender.com, accessed on 10 May 2023. (**B**) NP-p24 repartition in LNs without or (**C**) with poloxamers 4 h after injection. The NPs (BodipyFL, green) are localized next to B follicles (IgD, red) containing FDCs (CD35, blue). Lower panels represent zooms of the white squares presented in the upper panels. Scale bar 500 mm.

To map the dissemination of the NPs within secondary lymphoid organs, we performed imaging of the draining (inguinal left) and distant (axillary left and right) LN 4 h after subcutaneous injection of fluorescent NP (p24) or NP (p24)-Pol(24%) (Figure 5A). The results showed that in the absence of hydrogel Pol(24%), the NPs(p24) were not visible in the three observed LN. The residence of this liquid formulation at the injection site, as seen using tomography (Figure 4A,C), was then confirmed by the sectioning and immunolabelling of LN, showing no detectable presence of fluorescent NPs (Figure 5B). Moreover, the dispersion of the Pol(24%) formulation at distant sites was confirmed by their presence in both draining and distant LNs (Figure 5C).

### 2.6. Induction of Germinal Centers in Secondary Lymphoid Organs

**Figure 6:**
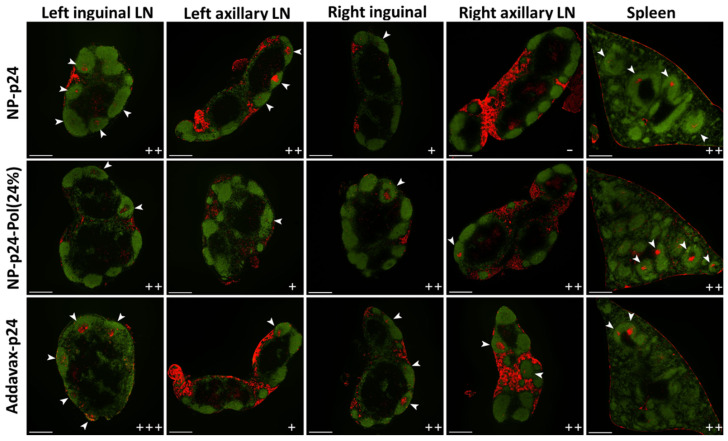
Germinal centers (GCs) in lymph nodes and spleen. B cell follicles were detected by immunostaining with IgD (green), and biotinylated peanut agglutinin (PNA) localized using DyLight 550 allowed visualization of GCs (red color and white arrowheads). The (+) and (−) symbols represent the number of GCs numbered in three independent lymph nodes/spleen for each condition. Scale bar, 500 µm.

The initiation of the adaptive immune response takes place in the secondary lymphoid organs. Depending on the traveling route from the injection site (lymphatics or bloodstream), vaccine formulation could migrate to either LNs or the spleen. The establishment of the adaptive immune response starts in GCs, which are central structures where the maturation of B lymphocytes occurs. The induction of GCs in LNs or the spleen is an indication of the initiation of an adaptive immune response. The NP-p24-Pol(24%) formulation induced the formation of GCs in both draining (inguinal left) and distant (axillary left and right) LNs (Figure 6). This dissemination of the vaccine-induced immune response is consistent with the distribution of fluorescent NPs observed 4 h after subcutaneous injection (Figure 5). Whereas the AddaVax^TM^ formulation followed the same repartition of activated LNs as the NP-p24-Pol(24%) formulation, the NP-p24 formulation only induced GCs in the draining LN (inguinal left) and the proximal LN (axillary left). No GCs were observed in the axillary right LN. All three formulations induced GCs in the spleen (Figure 6).

### 2.7. Induction of Specific p24 IgG-Antibodies

**Figure 7:**
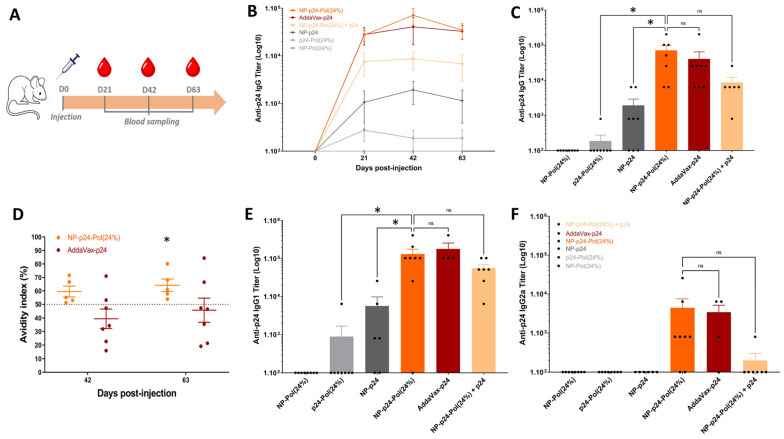
Antibody-induced immune response to different p24 vaccine formulations. (**A**) Immunization scheme of the one-shot immunization protocol. (**B**) Anti-p24 IgG antibody quantification at days 0, 21, 42, and 63 post-injection. (**C**) Anti-p24 IgG antibody quantification at day 42 post-injection. (**D**) Comparison of IgG avidity to p24 protein 42 days after injection of NP-p24-Pol(24%) and AddaVax-p24. (**E**) Quantification of the specific IgG1 and IgG2a (**F**) subclasses at day 42 post-injection. Each dot represents an animal. Data are presented as mean ± SEM and were statistically analyzed using one-way ANOVA followed by Tukey’s multiple comparison test (ns, not significant, * *p* < 0.1), except for (**D**), for which a one-sample *t*-test was performed (comparison to the theoretical mean of 50%).

To investigate if the discrepancies in the distribution of NP-p24 in LN according to the presence or absence of poloxamers could influence the initiation and intensity of the adaptive immune response, a quantification of the p24-specific antibody immune response was performed (Figure 7A). A single-shot immunization scheme with different NP-p24 formulations was performed with blood sampling 21, 42, and 63 days post-injection. The commercial adjuvant AddaVax^TM^ (MF59) was used as a reference. The anti-p24 IgG titers after administration of the formulation NP-p24 were significantly higher than the p24 directly incorporated into the Pol (without adsorption on an NP). The specific IgG titers after injection of NPp24-Pol(24%) formulation were as high as the titers obtained after administration of AddaVax-p24 (Figure 7B,C at 42 days). Interestingly, an intermediate formulation of NP-p24-Pol(24%) + p24 (half of the amount of p24 in the Pol(24%) hydrogel + half free) led to intermediate IgG titers comprised between NP-p24-Pol(24%) and NP-p24, highlighting an adjuvant function of the poloxamers (Figure 7B,C). Additionally, the avidity of the induced anti-p24 IgG 42 days post-injection was found to be similar for AddaVax^TM^ and NP-p24-Pol(24%) and slightly superior for the poloxamer formulation at day 63 post-injection when compared to the mean of 50% (Figure 7D).

The orientation of the adaptive immune response towards the humoral or cellular arm of immunity can be deciphered by the quantification of IgG subclasses. The induction of IgG1 pictures an antibody-based response whereas the induction of IgG2a is representative of a vaccine-induced cellular-based immune response. AddaVax^TM^ adjuvant was described as a balanced adjuvant activating both pathways and showed IgG1 and IgG2a induction (Figure 7E,F). A similarly balanced immune profile was determined for the NP-p24-Pol(24%) formulation, whereas the response to the NP-p24 vaccine predominantly relied on humoral antibody response, with only IgG1 detection [33] (Figure 7E,F).

## 3. Discussion

Poloxamers (trade names Pluronic^®^ or Kolliphor^®^ (pharma grade)) are synthetic, water-soluble, nonionic triblock copolymers formed by a central polar chain of polyoxypropylene (poly(propylene oxide), PPO) flanked by nonpolar chains of polyoxyethylene (poly(ethylene oxide), PEO). Poloxamers can undergo sol–gel transition with increasing concentration and temperature. This is due to a physical change from a dispersed nanostructured micelle state to a dense structure to form a thermosensitive hydrogel [34,35]. The rearrangement of poloxamers as micelles to a more structured network can be observed through the increase in the elastic (storage) modulus G′ and the viscous (or loss) modulus G″. The P188 and P407 concentrations play a major role in the production of a thermosensitive hydrogel, as only the 24% concentration led to the formation of a hydrogel (Figure 1). As the 15% concentration did not undergo sol–gel transition at the measured temperature (22–45 °C), this condition was used as a control of the presence of poloxamers in the formulations without physical rearrangement of poloxamer induced by changes in micellar properties [36].

To investigate the influence of poloxamers either in a dispersed micellar state (15%) or as a thermosensitive hydrogel (24%) on vaccine-induced immune response, we formulated a subunit vaccine by adsorbing a model protein Ag from HIV (p24) onto a PLA NP. The choice of poloxamers in this study was motivated by their known safety and because they are wildly used in the pharmaceutical industry. Poloxamers are not cytotoxic (Figure 3A) and are safe in mice when injected intramuscularly as described by Chen et al. [37]. The authors did not observe fibrotic regions in the liver or in the kidneys, abnormal body weight loss, or biochemical changes in the plasma of P188-vaccinated mice. After mixing these NP-p24 with the poloxamer solutions, we observed an increase in hydrodynamic diameter (Figure 2A) and a decrease in zeta potential (Figure 2B) for both concentrations, meaning that a corona of poloxamer was adsorbed on the surface of the NP-p24. Importantly, the p24 Ag was still available for antibody recognition after mixing with both poloxamer concentrations (Figure 2C). Additionally, the release of NP-p24 from the 24% hydrogel was slowed down by the presence of p24 at the surface of the NP, highlighting interactions between the protein and the poloxamers (Figure 2E). Seminal work from Blunk et al. described that poloxamers adsorb onto NPs with their hydrophobic part (PPO), whereas the hydrophilic part (PEO) is exposed at the NP’s surface, exposing the hydrophilic groups [38]. Modifications of the surface chemistry of NP (size, morphology, hydrophobicity…) could greatly influence their fate in terms of accumulation, targeting, circulating time, clearance by the immune system, barrier crossing, toxicity, or physical stability [39]. Therefore, we anticipated that the presence of poloxamers around the NPs would modify their biodistribution.

The subcutaneous injection of the 24% poloxamer hydrogel was expected to induce a depot effect at the injection site, as previously described for other thermogelling block copolymer gels [40]. Surprisingly, we observed the opposite, and a larger dissemination of NPs occurred when formulated with the 24% hydrogel compared to the formulation in PBS (Figure 4A–C). This result is consistent with previous results, which showed that 70% of subcutaneously injected NPs (polysterens beads with no coating) remained at the injection site for at least 24 h [41]. This observation was completed through the use of NP-p24 formulations in both 15% and 24% poloxamers, and the fluorescent NPs rapidly (4 h) escaped from the injection site (Figure 4E). These results highlighted the influence of the presence of poloxamers around the vaccine NPs rather than the hydrogel formation itself in the dissemination of the NP, as both formulations had the same biodistribution profile. Indeed, no depot effect was found but rather an anatomical distribution favorable to a large distribution and activation of draining and distant LNs. The injected fluorescent NPs carrying the Ag were detected in the draining inguinal left, axillary left, and right LNs 4 h after injection when combined with the 24% poloxamer solution (Figure 5A,B). The distribution of the fluorescent NPs in the LNs seemed to follow the subcapsular sinus, except in the draining (inguinal left) LN, where NPs were also observed within the B follicles. No signal was detected in the sampled LNs 4 h after injection in PBS, stressing the difference in the kinetics of distribution of NPs in the absence or presence of poloxamers. Similar results were obtained by Hawley et al., who observed an enrichment of the injected poly(lactic-co-glycolic) acid (PLGA) nanospheres in the regional LNs after subcutaneous injection [42]. A correlation between surface characteristics of NPs coated with poloxamers and their lymphatic distribution was highlighted, such as a surface coverage of 11–15% to minimize NP aggregation or a projected thickness of less than 3 nm for rapid and effective LN targeting [43]. It is known that the NPs’ characteristics influence their uptake by antigen-presenting cells and could favor LN targeting [14], but the addition of poloxamers at the NP surface facilitates the draining across the lymphatic vessels due to stabilizing PEO chains [17].

The wide distribution of NP-p24 into distant LNs in the presence of 24% poloxamer subsequently induced the formation of GCs, which are centers of activation of the adaptive immune response (Figure 6). Qualitative image analysis of the number of GCs in the four inguinal (left and right) and axillary (left and right) LNs revealed a similar distribution between NP-p24-Pol(24%) and Addavax^TM^. The NP-p24 formulation induced fewer GCs in the right LNs, which are more distant from the injection site (left leg). Splenic GCs were observed for all formulations, indicating partial migration of the Ag through the blood flow. The poloxamer formulation showed the ability to trigger GCs in all observed secondary lymphoid tissues and could be considered as a physical vaccine adjuvant, helping the Ag to widely disseminate. The use of poloxamer P188 as a vaccine adjuvant was reported previously and described as an immune modulation by repressing p38 phosphorylation and upregulating Th1 and Th2 immune response in splenocytes through significant activation of p38MAPK compared to Addavax^TM^ [37]. The use of P188 as an adjuvant for intramuscular vaccines was confirmed by a significant increase in neutralizing antibodies against SARS-CoV-2 RDB.

After assessment of antibody response following NP-p24-Pol(24%) injection, we can conclude that the presence of poloxamers in the formulation enhanced the immune response by (i) increasing the levels of specific IgGs compared to NP-p24 in PBS, (ii) increasing the avidity of IgG 9 weeks after injection compared to the standard Addavax^TM^, and (iii) inducing a more balanced Th1/Th2 response compared to NP-p24 (Figure 7). Then, the P188/P407 poloxamers showed adjuvant properties through the dissemination of the vaccine formulation without activation of PRR, similar to Addavax^TM^ (Figure 3B). Indeed, the use of a mouse macrophage reporter cell line (Raw-Blue™ 264.7) indicated the absence of activation of several PRR, such as TLRs, NOD receptors (NLRs), retinoic acid-inducible gene I (RIG-I)-like receptors (RLRs), or C-type lectin receptors (CLRs). However, the activation of innate receptors in other line cell lines would be interesting, as a poloxamer coating on NPs was shown to reduce uptake of NPs by macrophages [43]. Additionally, further studies on dose response of poloxamers on antibody induction would more precisely define the required amount of poloxamer, as P407 was found to induce transient hyperlipidemia in the blood after subcutaneous injection [40].

The improved adaptive immune response in the presence of poloxamers by multiple LN targeting could be an asset in vaccine development. Additionally, we showed that no gelation properties are required for a large lymphatic distribution, making industrial scale-up and storage at 4 °C possible. Finally, the addition of poloxamers in vaccine formulations could avoid multiple vaccine boosts due to them acting as both surfactants and adjuvants.

## 4. Materials and Methods

### 4.1. PLA Nanoparticles Synthesis

PLA NPs were brought to Adjuvatis (Lyon, France) and were produced using the nanoprecipitation technique as previously described [44]. Briefly, PLA was dissolved in acetone, and this organic solution was added dropwise to an aqueous solution under 250 rpm stirring. Organic solvents were then removed via evaporation under reduced pressure at 30 °C. The final PLA NP concentration was around 40 mg/mL and was measured by weighing the wet and dried materials.

### 4.2. Fluorescent PLA Nanoparticles Synthesis

The near-infrared fluorophore 1,1′-Dioctadecyl-3,3,3′,3′-Tetramethylindotricarbocyanine Iodide (DiR) was purchased from ThermoFisher Scientific (Waltham, MA, USA). The fluorescent PLA NPs (DiR, BodipyTR, or BodipyFL) were brought to Adjuvatis and were produced following the same method as described in [45] at a fluorophore:PLA concentration of 0.02% *w*/*w*.

### 4.3. p24 Ag Adsorption in PLA Nanoparticles

HIV-1 Gag p24 Ag was purchased and purified by PX’Therapeutics (Grenoble, France) from the Escherichia coli BL21 DE3 strain, and endotoxins were removed as previously described in [46]. The purity of p24 was higher than 97% as assessed by silver-nitrate-stain-reducing SDS-PAGE. The endotoxin content was lower than 5 EU/mg of p24 protein, as determined using the QCL-1000 Quantitative Chromogenic Limulus Amebocyte Lysate (LAL) kit (BioWhittaker, Walkersville, Verviers, Belgium). PBS 1X (PBS) Gibco was purchased from Sigma-Aldrich. The p24 protein was diluted in PBS at 400 μg/mL. PLA NP were diluted at a concentration of about 20 mg/mL in PBS and 1 volume was added to 1 volume of the protein solution. The adsorption reaction was incubated for 2 h at room temperature (RT) with moderate end-overhead stirring. The unbound p24 protein was collected in the supernatant via centrifugation at 10,000× *g* for 10 min and quantified using a µ-BCA kit (Protein Assay, Thermo Scientific, Waltham, MA, USA). The absorbance of the samples was measured at 562 nm using a microplate reader (Multiskan FC, Thermo Scientific).

### 4.4. PLA Nanoparticles Characterization

The hydrodynamic diameter and size distribution (Polydispersity index, PdI) were determined via dynamic light scattering (DLS) at a temperature of 25 °C and a scattering angle of 173° using a Zetasizer Nano ZS (Malvern, UK). The formulations were diluted in 1 mM NaCl 0.22 µm filtered solution, and each value was the mean of four independent measurements. The electrophoretic mobilities were measured using the same equipment and the same highly diluted samples but at an angle of 12.8°. The measurements were converted to zeta potential according to Smoluchowski’s equation.

### 4.5. P407/P188 Hydrogel Formulations Preparation

Poloxamer P407 (MW 13,971 g/mol; 72.5% PEO) and poloxamer P188 (MW 8845 g/mol; 81.0% PEO) were obtained from BASF (Ludwigshafen, Germany). Hydrogels were prepared according to the “cold method”. Briefly, the appropriate amounts of P407 and P188 for each formulation were carefully weighed and placed in a vial. The required volume of PBS precooled at 4 °C was added slowly to the vial. The dispersion was stored under agitation (200 rpm) on ice until the poloxamers were dissolved completely. All the samples were prepared on a *w*/*v* ratio basis.

### 4.6. PLA Nanoparticles Incorporation in Hydrogel Formulations

The appropriate volume of blank NP or loaded NP was added to the already-formed hydrogel at 4 °C. The formulation was kept under magnetic stirring at 200 rpm for 2 h to ensure the homogeneity of the final solution without compromising the integrity of the NP. The formulations were then stored at 4 °C until used. All the samples were prepared on a *w*/*v* basis.

### 4.7. Rheological Analysis

Rheological analysis was performed using a nondestructive and contact-free viscoelastic testing instrument (ElastoSens™ Bio, Rheolution Inc., La Rochelle, France). After pouring 5 mL of each poloxamer formulation (15 and 24%) into cylindrical sample holders, viscoelastic measurements were conducted using a temperature ramp from 22 to 45 °C at a heating rate of 1 °C/min with one recording point every 3 s. All measurements were performed in triplicate. The sol–gel transition temperature (T_sol/gel_) was determined from the temperature obtained when G′ was halfway between the values for the solution and for the gel [47].

### 4.8. In Vitro Erosion Profiles of P407/P188 Hydrogel Formulations

Hydrogel erosion profiles were obtained following the method described by Zhang et al. [48]. Briefly, 2.5 mL of cold formulations (hydrogel and hydrogel–NP) were transferred into preweighted glass vials with a diameter of 2.4 cm and incubated at 37 °C. After the formulations were gelled, 5 mL of PBS pre-equilibrated at 37 °C were carefully layered over the surface of the hydrogel. At determined time points, the PBS supernatant was removed. The remaining hydrogel in the vial was dried overnight in a vacuum, and the residual weight of the hydrogel was determined. The percentage of mass loss (% *w*/*w*) was calculated using the equation as follows:

W_loss_ = 1 − (W_t_ − W_0_)/((W_hydrogel_ − W_0_) × 0.24)

where (W_t_) is the experimental weight of the dried remaining hydrogel at each time, (W_0_) is the initial weight of the vial without hydrogel, (W_hydrogel_) is the experimental weight of the vial and hydrogel at the beginning of the experiment, and (0.24) is the dry weight of the poloxamers added to prepare the hydrogel. The amount of hydrogel dissolved during the study period was calculated by the weight difference of the vial. The erosion profile was then obtained by plotting the cumulative weight of each hydrogel formulation dissolved versus time. The entire experiment was performed in triplicate.

### 4.9. Release of NPs from the Pol(24%) Hydrogel

The release kinetics of NP from the hydrogel was evaluated using fluorescent NPs (BodipyTR). The NP(BodipyTR)-Pol(24%) formulations (with or without p24) were incubated for one hour at 37 °C to achieve the sol–gel transition. Then, a quantity of PBS equal to half the volume of the hydrogel was added to the top of the hydrogel. Aliquots of PBS were taken every hour and replaced by an equal volume of PBS. The quantification of the fluorescent NP release was assessed by spectrophotometry (TECAN Infinite 2000).

### 4.10. In Vitro Toxicity Profile and Immune Activation

Immortalized DC2.4 (murine bone marrow-derived dendritic cells) cells were obtained from InvivoGen (Toulouse, France). They were cultured in RPMI-1640 medium, supplemented with 10% heat-inactivated fetal bovine serum (FBS), 10 mM Hepes and 50 µM β-mercaptoethanol. Cells were cultured in a 37 °C incubator under 5% CO_2_ and 95% humidity. Cytotoxicity was evaluated 24 h after the addition of formulations (2% NP, 20µg/mL p24) with a ratio of 10,000 NP per cell. Cell viability was assessed using a PrestoBlue™ Assay (Thermo Scientific, Waltham, MA, USA) according to the manufacturer’s instructions. Briefly, 11 µL of PrestoBlue™ Cell Viability Reagent was added to the wells, and plates were incubated for 20 min at 37 °C. Fluorescence was detected on a Tecan i-control Infinite M1000 (560 nm/590 nm; bandwidth 10 nm) instrument (Tecan, Männedorf, Switzerland). Fluorescence was determined as the mean of three replicates and three independent experiments.

The reporter macrophage cell line (RAW-Blue cells; InvivoGen, San Diego, CA, USA) is derived from murine RAW 264.7. Macrophages secreted embryonic alkaline phosphatase (SEAP) after activation by NF-κB and activator protein 1 (AP-1). The macrophage reporter cells express toll-like receptors (TLRs) and NOD-like, RIG-I-like, and C-type lectin receptors. Activation of these receptors induces signaling pathways, leading to the activation of NF-κB and AP-1 and the subsequent production of SEAP. Cells were grown to 80% confluence in Dulbecco’s modified Eagle’s medium Glutamax (DMEM) supplemented with 10% heat-inactivated FBS and 200 μg/mL Zeocin antibiotic (InvivoGen) at 37 °C in 5% CO_2_.

The quantification of the activation of RAW-Blue™ cells was performed following the manufacturer’s recommendation. Cells (150,000 cells/well) were seeded in a 24-well flat-bottom plate. Formulations were added (2% NP, 20 µg/mL p24) in triplicate and incubated for 18 h at 37 °C and 5% CO_2_. Media was considered as the only negative control, and lipopolysaccharide (LPS, ThermoFisher Scientific) was tested as the positive control at a concentration of 5 mg/mL. Subsequently, a 1/10 dilution of cell supernatants in QUANTI-Blue solution (Invivogen) was incubated for 2 h at 37 °C and 5% CO_2_. The SEAP levels were quantified using a microplate reader (Tecan, Männedorf, Switzerland) at 620 nm.

### 4.11. In Vivo Biodistribution Study

SKH1 male mice were bred at Charles River Laboratories (L’Arbresle, France) and housed at the P-PAC-specific pathogen-free animal facility of the Centre Léon Bérard, Lyon, France. All of the experiments were performed in accordance with animal welfare regulations for their use for scientific purposes governed by European Directive 2010/63/EU. Protocols were validated by the local Animal Ethics Evaluation Committee (CECCAPP: C2EA-15) and authorized by the French Ministry of Education and Research.

Seven-week-old mice received a single subcutaneous injection of 0,96 µM DiR/mouse diluted in the different formulations: free DiR in 24% hydrogel (DiR-Pol(24%)), NP-DiR suspension (NP(DiR)), NP(DiR) in 24% hydrogel (NP(DiR)-Pol(24%)) (n = 5 mice per group). Whole-body fluorescence and injection site fluorescence were recorded 5 min; 4 h; and 1, 7, 17, 24, 30, and 42 days after injection using the FMT4000 fluorescence tomography imaging system (Perkin Elmer, Waltham, MA, USA). Prior to imaging, mice were placed in an induction box to be anesthetized using isoflurane gas (3%) and positioned in the FMT system imaging chamber. Filter sets were chosen with the following parameters for DiR: excitation at 750 nm and emission at 782 nm. Acquired images were analyzed with TrueQuant software version 4.0. The quantities of DiR in the whole body and at the injection site were quantified by drawing regions of interest (ROI).

### 4.12. In Vivo Immunological Study

CB6F1 female mice were bred at Charles River Laboratories (L’Arbresle, France) and housed at the pathogen-free animal facility Plateau de Biologie Expérimentale de la Souris (PBES, Lyon, France). All of the experiments were performed in accordance with animal welfare regulations for their use for scientific purposes governed by European Directive 2010/63/EU. Protocols were validated by the local Animal Ethics Evaluation Committee (CECCAPP: C2EA-15) and authorized by the French Ministry of Education and Research.

Mice were divided into 6 groups of 8 animals. Eight-week-old mice received a single subcutaneous injection of 1 µg p24/mouse diluted in the different formulations: free p24 in 24% hydrogel (p24-Pol(24%)), NP-p24, NP-p24 in 24% hydrogel (NP-p24-Pol(24%)), and NP-p24-Pol(24%) with free p24 (NP-p24-Pol(24%) + p24), with a total amount of 1 µg of p24, i.e., 0.5 µg p24 onto NPs and 0.5 µg of free p24. The negative control was composed of NP in 24% hydrogel (NP-Pol(24%)). The standard used as a positive control was 50 µL of AddaVax^TM^, a MF-59-like squalene adjuvant (Invivogen, Toulouse, France), in accordance with the supplier’s recommendations. Mice were bled retro-orbitally (100 µL) before immunization (D0) and at days 21, 42, and 63 after the immunization to monitor Ab response. The samples were heated at 37 °C for 30 min, then centrifuged twice at 10,000× *g* for 10 min, and sera were stored at −20 °C for further analysis.

### 4.13. Lymph Node and Spleen Imaging

CB6F1 female mice were bred at Charles River Laboratories (L’Arbresle, France) and housed at the pathogen-free animal facility Plateau de Biologie Expérimentale de la Souris (PBES, Lyon, France).

To map the distribution of NPs in the draining and distant LNs, three mice of each group were sacrificed 4 h post-immunization with NP(Bodipy-FL)-p24 or NP(Bodipy-FL)-p24-Pol(24%) to harvest inguinal and axillary LN. Organs were snap frozen in Optimal Cutting Temperature compound (Tissue-Tek O.C.T, Sakura Finetek, Torrance, CA, USA) and stored at −80 °C until cryosection. Serial longitudinal sections, 40 μm thick, were collected through each LN using a cryostat microtome (Leica Biosystems, Nußloch, Germany). Sections were incubated with rat anti-mouse IgD conjugated with Alexa Fluor 594 (Biolegend, San Diego, CA, USA) to detect B cells follicles and with anti CD35 conjugated with BV421 (BD Optibuild, San Jose, CA, USA) to detect follicular dendritic cells (FDCs). Pictures were acquired with a high-content screening inverted confocal microscope (CQ1, Yokogawa, Lyon, France)

For the induction of GCs in secondary lymphoid organs, three mice of each group were sacrificed 21 days post-immunization with NP-p24, NP-p24-Pol(24%), or AddaVax-p24 to harvest both inguinal LNs, both axillary LNs, and the spleen. Organs were snap frozen in Optimal Cutting Temperature compound (Tissue-Tek O.C.T, Sakura Finetek, Torrance, CA, USA) and stored at −80 °C until cryosection. Serial longitudinal sections 8 μm thick were collected through each LN using a cryostat microtome (Leica Biosystems, Nußloch, Germany). The central section and the two sections spaced 250 µm apart were fixed on glass slides with acetone. Similarly, the transversal section was collected in the large part of the spleen and fixed with acetone. Sections were first incubated with rat anti-mouse IgD primary antibody (Biolegend, San Diego, CA, USA) to detect B cell follicles and with biotinylated peanut agglutinin (PNA) (Vectors Laboratories, Burlingame, CA, USA) to detect GCs. They were then incubated with goat anti-rat IgG secondary antibody conjugated with Alexa Fluor 488 (Thermo Scientific, Rockford, IL, USA) to reveal anti-IgD (green) and with streptavidin conjugated with DyLight 550 (Thermo Scientific, Rockford, IL, USA) to reveal biotinylated PNA (red). Slides were visualized and acquired using a Nikon Eclipse Ti-E inverted microscope (Nikon Instruments Inc., Melville, NY, USA) equipped with a 4× objective. Measurements of B cells and GCs areas were carried out using Image J software, version 1.52p (U.S. National Institutes of Health, Bethesda, MD, USA). GCs were manually counted. The score (−, +, ++, or +++) was established according to the number and the size of GCs relative to B cell follicles.

### 4.14. Specific Anti-p24 Antibody Quantification and Avidity Assay

Serum samples were analyzed for the detection of p24-specific IgG via enzyme-linked immunosorbent assay (ELISA). Ninety-six-well Nunc maxisorp plates were coated with 100 μL of 1 μg/mL p24 protein overnight at RT. The excess p24 was eliminated, and plates were blocked with 10% non-fat dry milk for 1 h at 37 °C to prevent non-specific binding of the Abs. Plates were washed with PBS/0.05% Tween-20 (PBST) using an automatic plate washer (ThermoScientific wellwash Versa). Sera were serially diluted in PBS/BSA 1%. Then, 100 μL of each sample was incubated on blocked plates for 1 h at 37 °C. Horse radish peroxidase (HRP)-coupled goat anti-mouse antibodies (IgG, IgG1 and IgG2A, all purchased from Southern Biotech) were diluted at 0.1 µg/mL in PBS/BSA 1% and added into the wells after washing. After 1 h at 37 °C, wells were washed three times. Finally, antibodies coupled to HRP were revealed using 100 µL of reconstituted TMB substrate reagent (BD Bioscience, #555214), and reactions were stopped 30 min later with 1 N sulfuric acid (VWR, #32053.602). The optical density (OD) at 450 nm with a correction at 620 nm was measured using a microplate reader (Multiskan FC, Thermo Scientific).

The avidity of serum immunoglobulins was determined using the Ab–Ag binding resistance to 8 M urea. Samples were prediluted to give an OD at 450 nm (OD450) readout of between 0.7 and 3.0 and were added to p24-coated plates. The plates were then washed three times with either PBST or 8 M urea in PBST before incubation with anti-mouse IgG-HRP. Samples were developed with TMB as described above. The avidity index (in percent) was calculated as the OD450 of urea-treated samples/OD450 of PBST-treated samples. Antisera with index values exceeding 50% were ascribed a high avidity, those with index values of 30 to 50% were ascribed intermediate avidity, and those with index values of <30% were ascribed a low avidity.

### 4.15. Statistical Analysis

Statistical analyses were performed using GraphPad Prism Version 6.0 software. All of the data are presented as the mean ± SD, except for Figure 7, in which SEM was used due to interanimal variability. One- or two-way ANOVA tests were run followed—if indicated—by multiple comparison tests: Tukey’s test was used for the comparison of every mean, and Dunnett’s test was used for the comparison of every mean with the control mean. For Figure 7D, a one-sample *t*-test was performed to compare the experimental mean to the theoretical mean of 50. Significance level is indicated as * *p* < 0.05, ** *p* < 0.01, *** *p* < 0.001, **** *p* < 0.0001.

## Figures and Tables

**Table 1:** Physicochemical characterization of different formulations (hydrodynamic diameter, polydispersity index (PDI), and zeta potential). NP: nanoparticles; Pol(X%): poloxamer at a concentration of X%; SD: standard deviation.

Formulation	Hydrodynamic Diameter ± SD (nm)	PDI ± SD	Zeta Potential ± SD (mV)
NP	194 ± 2.42	0.02 ± 0.01	−54 ± 0.74
NP-p24	197 ± 0.97	0.02 ± 0.01	−64 ± 0.95
NP-p24-Pol(15%)	214 ± 2.06	0.04 ± 0.01	−16 ± 0.33
NP-p24-Pol(24%)	221 ± 2.50	0.03 ± 0.01	−15 ± 0.40

## Data Availability

The data presented in this study are available on request from the corresponding author.
